# A transnasal traction method using a novel traction device in pharyngeal endoscopic submucosal dissection

**DOI:** 10.1055/a-2174-7050

**Published:** 2023-10-06

**Authors:** Yuhei Umeda, Yasuhiko Hamada, Yohei Ikenoyama, Hiroki Yukimoto, Misaki Nakamura, Noriyuki Horiki, Hayato Nakagawa

**Affiliations:** Department of Gastroenterology and Hepatology, Mie University Hospital, Tsu, Japan

Pharyngeal endoscopic submucosal dissection (ESD) is technically challenging because of the narrow and complex space involved in endoscopic maneuverability. Thus, the traction technique is important for completing the procedure. The transnasal endoscope method or transoral forceps method was useful for creating good countertraction during pharyngeal ESD 1 2 . However, the former method requires another endoscope, and the latter method may interfere with the transoral endoscopic maneuver. The EndoTrac (Top Corporation, Tokyo, Japan) can improve the submucosal layer’s visibility by changing the traction direction during ESD ( Fig. 1 ) 3 4 5 .

**Fig. 1:**
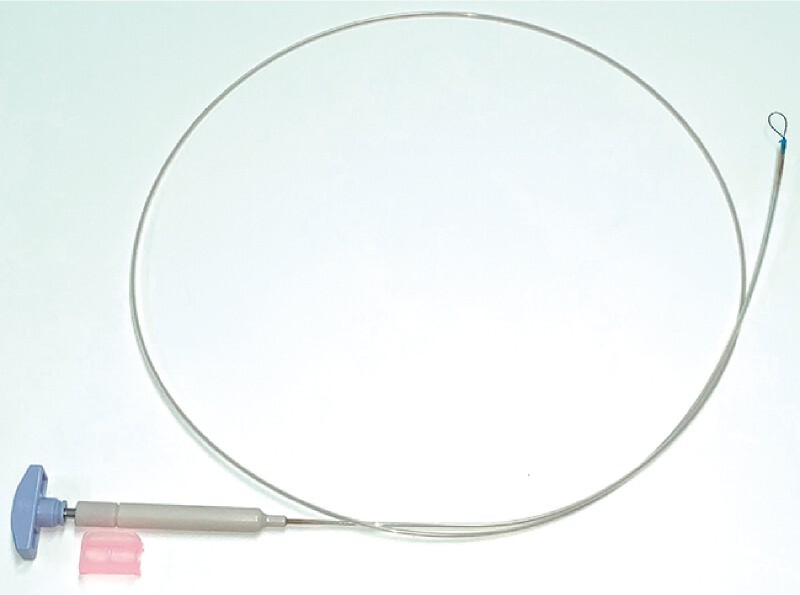
The EndoTrac.

A 71-year-old man with a history of endoscopic resection for superficial esophageal cancer underwent follow-up endoscopy, which detected a superficial hypopharyngeal cancer extending from the posterior hypopharynx to the esophageal orifice. ESD was performed under general anesthesia ( Video 1 ). The lesion was marked circumferentially ( Fig. 2 ), and a circumferential mucosal incision was made. Subsequently, the EndoTrac was inserted nasally into the mouth, and the device tip was grasped and drawn out of the mouth using a transoral endoscope. The EndoTrac was then tied to an endoclip and re-inserted into the mouth. The tip with an endoclip was deployed on the oral side of the lesion margins ( Fig. 3 ), and the device end was pulled nasally to optimize visibility of the subepithelial layer. Good countertraction was obtained, and the endoscopic maneuver did not interfere with the EndoTrac ( Fig. 4 ). The lesion was resected en bloc within 100 minutes without adverse events ( Fig. 5 ). The resected specimen pathologically revealed squamous cell carcinoma in situ (WHO classification). Curative resection was achieved, and endoscopic follow-up was performed without further treatments. Post-procedural stenosis and cancer recurrence were not observed 6 months after the ESD.

**Video 1** A transnasal traction method using a novel traction device in pharyngeal endoscopic submucosal dissection.


**Fig. 2:**
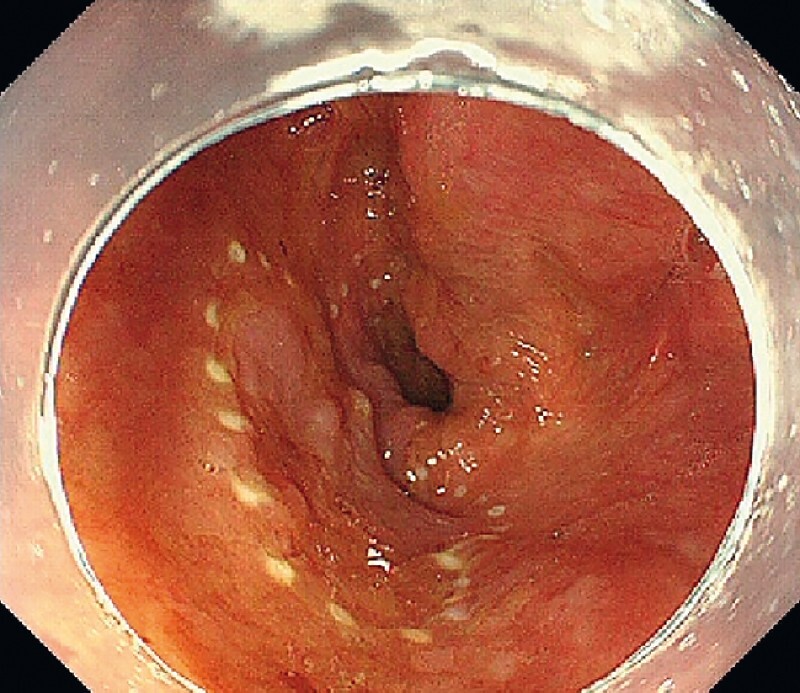
The lesion was marked circumferentially.

**Fig. 3:**
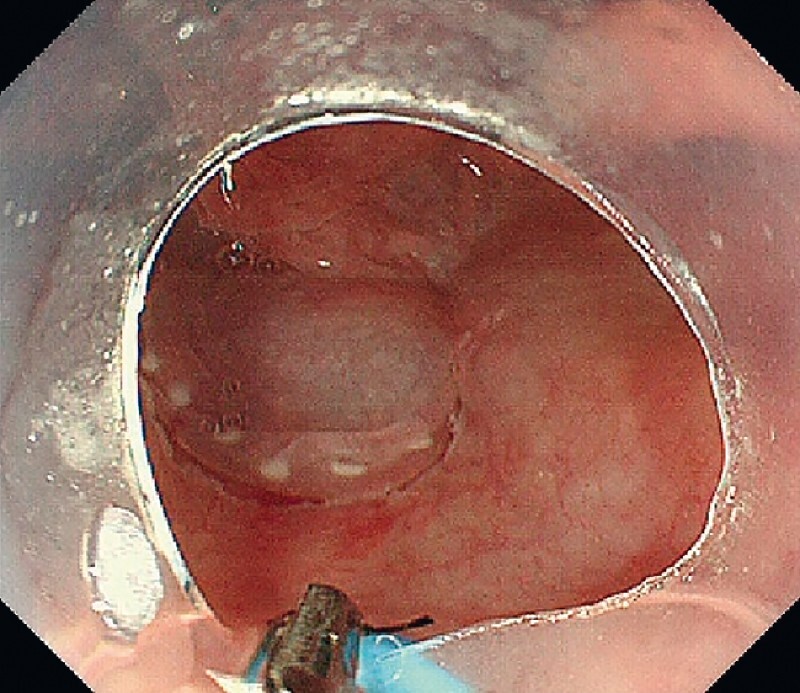
Endoscopic view before deploying the EndoTrac.

**Fig. 4:**
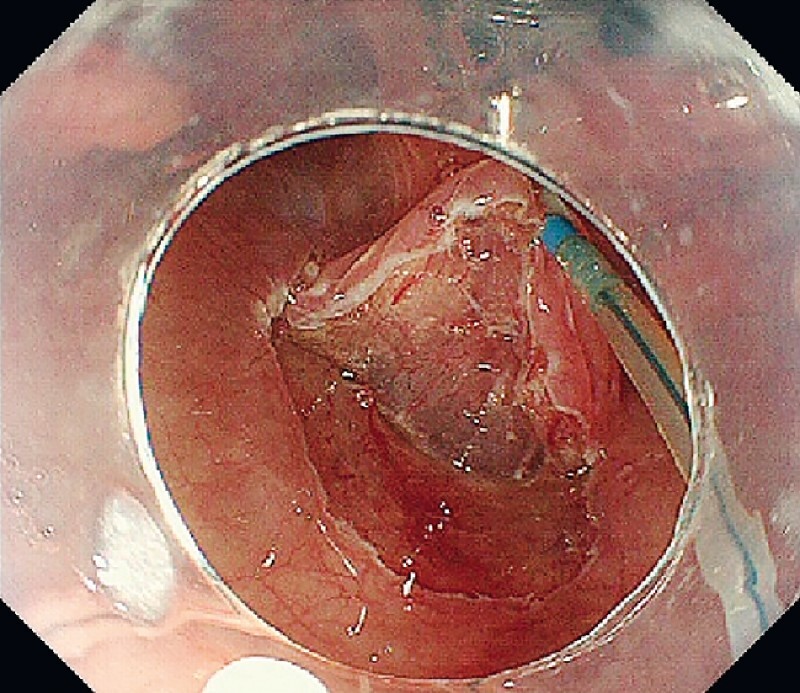
The EndoTrac facilitated adequate traction and improved the visibility of the cutting line.

**Fig. 5:**
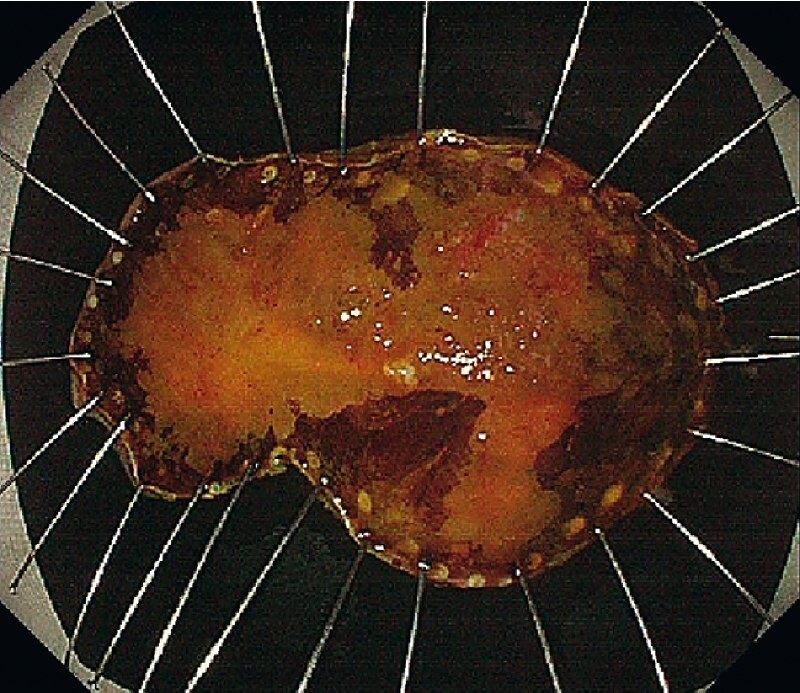
The lesion was resected en bloc without adverse events. The resected specimen measured 60 × 25 mm, with a lesion size of 40 × 15 mm.

Transnasal traction using the EndoTrac can create good countertraction and facilitates a well-visualized cutting layer without interference from transoral endoscopic maneuvers during pharyngeal ESD.

Endoscopy_UCTN_Code_TTT_1AO_2AG

## Notes

Endoscopy E-Videos

https://eref.thieme.de/e-videos
*E-Videos* is an open access online section of the journal *Endoscopy* , reporting on interesting cases and new techniques in gastroenterological endoscopy. All papers include a high-quality video and are published with a Creative Commons CC-BY license. Endoscopy E-Videos qualify for HINARI discounts and waivers and eligibility is automatically checked during the submission process. We grant 100% waivers to articles whose corresponding authors are based in Group A countries and 50% waivers to those who are based in Group B countries as classified by Research4Life (see: https://www.research4life.org/access/eligibility/ ). This section has its own submission website at https://mc.manuscriptcentral.com/e-videos
